# Injuries Associated With Standing Electric Scooter Use

**DOI:** 10.1001/jamanetworkopen.2018.7381

**Published:** 2019-01-25

**Authors:** Tarak K. Trivedi, Charles Liu, Anna Liza M. Antonio, Natasha Wheaton, Vanessa Kreger, Anna Yap, David Schriger, Joann G. Elmore

**Affiliations:** 1 Veterans Administration, Greater Los Angeles Healthcare System, Los Angeles, California; 2 National Clinician Scholars Program, University of California, Los Angeles, Los Angeles, California; 3 Department of Emergency Medicine, University of California, Los Angeles; 4 Department of Surgery, Stanford University, Stanford, California; 5 Department of Surgery, University of California, Los Angeles; 6 Office of Health Informatics and Analytics, UCLA Health, University of California, Los Angeles; 7 Division of General Internal Medicine and Health Services Research, University of California, Los Angeles

## Abstract

**Importance:**

Since September 2017, standing electric scooters have proliferated rapidly as an inexpensive, easy mode of transportation. Although there are regulations for safe riding established by both electric scooter companies and local governments, public common use practices and the incidence and types of injuries associated with these standing electric scooters are unknown.

**Objective:**

To characterize injuries associated with standing electric scooter use, the clinical outcomes of injured patients, and common use practices in the first US metropolitan area to experience adoption of this technology.

**Design, Setting, and Participants:**

This study of a case series used retrospective cohort medical record review of all patients presenting with injuries associated with standing electric scooter use between September 1, 2017, and August 31, 2018, at 2 urban emergency departments associated with an academic medical center in Southern California. All electric scooter riders at selected public intersections in the community surrounding the 2 hospitals were also observed during a 7-hour observation period in September 2018.

**Main Outcomes and Measures:**

Incidence and characteristics of injuries and observation of riders’ common use practices.

**Results:**

Two hundred forty-nine patients (145 [58.2%] male; mean [SD] age, 33.7 [15.3] years) presented to the emergency department with injuries associated with standing electric scooter use during the study period. Two hundred twenty-eight (91.6%) were injured as riders and 21 (8.4%) as nonriders. Twenty-seven patients were younger than 18 years (10.8%). Ten riders (4.4%) were documented as having worn a helmet, and 12 patients (4.8%) had either a blood alcohol level greater than 0.05% or were perceived to be intoxicated by a physician. Frequent injuries included fractures (79 [31.7%]), head injury (100 [40.2%]), and contusions, sprains, and lacerations without fracture or head injury (69 [27.7%]). The majority of patients (234 [94.0%]) were discharged home from the emergency department; of the 15 admitted patients, 2 had severe injuries and were admitted to the intensive care unit. Among 193 observed electric scooter riders in the local community in September 2018, 182 (94.3%) were not wearing a helmet.

**Conclusions and Relevance:**

Injuries associated with standing electric scooter use are a new phenomenon and vary in severity. In this study, helmet use was low and a significant subset of injuries occurred in patients younger than 18 years, the minimum age permitted by private scooter company regulations. These findings may inform public policy regarding standing electric scooter use.

## Key Points

**Question:**

What are the types of injuries associated with standing electric scooter use and the characteristics and behaviors of injured patients?

**Findings:**

In this study of a case series, 249 patients presented to the emergency department with injuries associated with electric scooter use during a 1-year period, with 10.8% of patients younger than 18 years and only 4.4% of riders documented to be wearing a helmet. The most common injuries were fractures (31.7%), head injuries (40.2%), and soft-tissue injuries (27.7%).

**Meaning:**

In this study, injuries associated with electric scooter use were common, ranged in severity, and suggest low rates of adherence to existing regulations around rider age and low rates of helmet use.

## Teaser

This study of a case series investigates injuries associated with standing electric scooter use, characteristics and outcomes of injured patients, and common use practices of scooters in a US city.

## Introduction

Standing electric scooters first appeared in Santa Monica, California, in September 2017, when the micromobility company Bird Rides, Inc, placed thousands of their scooters all around the city.^1^ These scooters were immediately popular with riders, presumably due to their ease of use, convenience, and low cost. The scooters are located and unlocked using a downloaded smartphone application, rides are paid for by the minute, and the ride can be ended anywhere the rider decides. With a maximum speed of 15 mph,^2^ these short-range electric vehicles consist of a narrow platform on which the rider stands with 1 foot in front of the other and a waist-high rod with handlebars for steering; after kicking off initially with 1 foot, riders accelerate and brake the scooter using triggers activated with their thumbs.

Companies offering standing electric scooters are rapidly expanding in the United States. For example, Lime-S scooters are available in more than 60 US cities and 6 cities internationally,^3^ and in April 2018, Bird Rides, Inc, announced more than 1 million completed rides.^4^ Today, several major companies, including Bird and Lime, offer dockless electric scooter services, and several other companies, including the ride-sharing companies Uber and Lyft, have recently entered the market.^5^ Availability is projected to grow rapidly, with market analysis showing that Lime was valued at $1.1 billion and its rival Bird was valued at more than $2 billion.^6^

The early personal transporters by Segway, introduced in 2001, were few in number, expensive to use, restricted to tourist locations, and associated with a specific set of injuries.^7^ In comparison, many thousands of riders are now using standing electric scooters daily on US streets shared with millions of pedestrians and drivers. Therefore, understanding the impact of rising scooter use on public health is more important than ever. Local laws regarding electric scooters are variable, with most locales prohibiting riding on the sidewalk and requiring the use of helmets,^8^ but no uniform set of policies exists, and differences in enforcement further amplify this variation. The scooter rental smartphone applications require riders to state that they will comply with state and local laws, show proof of a driver’s license, be older than 18 years, and use a helmet as part of their initial user agreements, but it is unclear to what extent these requirements are followed. Debates over the role of greater regulation of electric scooters continue in cities like San Francisco^9^ and Santa Monica, California.^10^ Of note, a bill supported by Bird to remove the helmet requirement for riders aged 18 years and older was recently signed into law in California,^11,12^ illustrating the timeliness of this issue as well as the importance of garnering evidence to guide policy.

Given our institution’s proximity to where these electric scooters were first available in the United States, we have the unique ability to describe injuries associated with electric scooters that were severe enough to trigger an emergency department (ED) visit over the course of 1 year. We report on the patient demographic and clinical characteristics of injuries associated with electric scooter use evaluated in our institution’s 2 EDs. Additionally, we conducted public observations to describe common scooter riding practices in the community near the 2 EDs.

## Methods

### Study Design

We retrospectively analyzed deidentified data from all patient encounters for standing electric scooter injuries presenting to either of 2 EDs affiliated with the University of California, Los Angeles (UCLA), Ronald Reagan UCLA Medical Center and UCLA Medical Center–Santa Monica. We report summary statistics on the continuous and categorical variables of interest. Additionally, we observed a convenience sample of scooter riders to describe common use practices of standing electric scooters in the community surrounding our hospitals (eAppendix in the Supplement). The UCLA institutional review board approved all aspects of this study with waiver of informed patient consent. The study was conducted using the Strengthening the Reporting of Observational Studies in Epidemiology (STROBE) reporting guideline.^13^

### Data Collection

We identified all ED encounters for injuries associated with standing electric scooter use in patients of any age by querying our unified electronic medical record for ED encounters between September 1, 2017, and August 31, 2018, that contained a clinician note with any of the non–case-sensitive terms “scooter,” “bird,” or “lime.” Two of us (T.K.T. and C.L.) reviewed the medical records to verify eligibility and excluded ED encounters that were not due to trauma associated with standing electric scooter use. The eAppendix in the Supplement describes our process of determining inclusion and data abstraction, and eTable 1 in the Supplement details how categories of injuries were assigned using *International Classification of Diseases, Ninth Revision, Clinical Modification *(*ICD-9-CM*) diagnosis codes.

### Statistical Analysis

In this descriptive study of a case series, we report proportions, calculate means and standard deviations for normally distributed data, and calculate medians and interquartile ranges for data that were not normally distributed.

## Results

Two hundred forty-nine patients (145 [58.2%] male; mean [SD] age, 33.7 [15.3] years) presented to the emergency department with injuries associated with standing electric scooter use during the study period (Figure; eFigure in the Supplement). The demographic and incident characteristics of these patients are shown in Table 1. A majority of patients (152 [61.0%]) were between the ages of 18 and 40, although ages ranged from 8 to 89, and 27 patients (10.8%) were younger than 18 years. Of the 249 patients, 228 (91.6%) were riders and 21 (8.4%) were nonrider pedestrians (11 hit by a scooter, 5 tripped over a parked scooter, and 5 were attempting to lift or carry a scooter not in use). A majority of ED visits (141 [56.6%]) occurred during the late afternoon and evening hours, between 3 pm and 11 pm.

**Figure.**
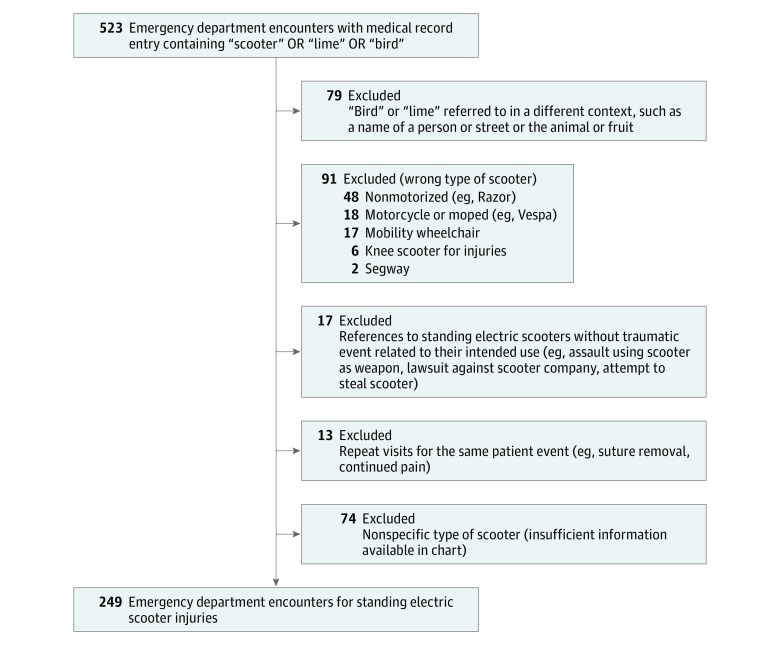
Identifying Visits for Injuries Associated With Standing Electric Scooter Use

**Table 1.** Patient and Accident Characteristics for ED Visits Associated With Standing Electric Scooters During a 1-Year Period

Characteristic	No. (%)		
	Riders (n = 228)	Nonriders (n = 21)	Total (N = 249)
**Demographic Characteristics**			
Age, y			
<18	26 (11.4)	1 (4.8)	27 (10.8)
18-25	61 (26.8)	1 (4.8)	62 (24.9)
26-40	85 (37.3)	5 (23.8)	90 (36.1)
41-64	51 (22.4)	10 (47.6)	61 (24.5)
≥65	5 (2.2)	4 (19.1)	9 (3.6)
Male	134 (58.9)	11 (52.4)	145 (58.2)
**Accident Characteristics**			
Mechanism of injury			
Rider			
Fall, no specific details	183 (80.2)	NA	NA
Collision with an object	25 (11.0)	NA	NA
Hit by a vehicle or moving object	20 (8.8)	NA	NA
Nonrider			
Hit by scooter	NA	11 (52.4)	NA
Tripped over scooter in road	NA	5 (23.8)	NA
Other^a^	NA	5 (23.8)	NA
Mechanism of ED transport			
Self-presented	151 (66.2)	17 (81.0)	168 (67.5)
Emergency medical services	77 (33.8)	4 (19.1)	81 (32.5)
Emergency medical services trauma activation	20 (8.8)	0	20 (8.0)
Time of day			
7 am-3 pm	57 (25.0)	8 (38.1)	65 (26.1)
3 pm-11 pm	130 (57.0)	11 (52.4)	141 (56.6)
11 pm-7 am	41 (18.0)	2 (9.5)	43 (17.3)
Helmet use^b^			
Unknown	144 (63.2)	NA	NA
No helmet	74 (32.5)	NA	NA
Wearing a helmet	10 (4.4)	NA	NA
Drug or alcohol intoxication^c^			
Blood alcohol level >0.05% or subjectively indicated by physician	12 (5.2)	0	12 (4.8)

Abbreviations: ED, emergency department; NA, not applicable.

^a^ Other mechanisms involved 4 people injuring foot while attempting to lift or manipulate scooter and 1 person who injured their hand while trying to lift scooter.

^b^ Numbers for nonriders are not calculated, as they would not be wearing helmets. One nonrider was a bicyclist wearing a helmet who was hit by a scooter.

^c^ Patients were considered not intoxicated unless there was physician documentation of intoxication or blood alcohol testing with a result of greater than 0.05%.

Among scooter riders, the most common mechanisms of injury were fall (183 riders [80.2%]), collision with an object (25 riders [11.0%]), and being hit by a moving vehicle or object (20 riders [8.8%]). Only 10 riders were documented as wearing a helmet, constituting 4.4% of all riders or 11.9% of riders whose helmet use status was documented. Twelve patients (4.8%) had physician-documented intoxication or a blood alcohol level greater than 0.05%.

Table 2 describes the ED evaluation and injury characteristics of patients presenting with injuries associated with standing electric scooter use. The majority of patients (200 [80.3%]) received imaging in the ED, with the most common imaging studies being radiographs or computed tomography of the distal upper extremity (36.5%), computed tomography of the head (29.7%), and radiographs or computed tomography of the distal lower extremity (20.1%). A total of 8.4% of patients underwent a trauma-protocol computed tomography scan (head, cervical spine, chest, abdomen, and pelvis), indicating high concern for serious injury. Two hundred thirty-four patients (94.0%) were discharged home from the ED.

**Table 2.** Emergency Department Resource Use and Injury Characteristics

Characteristic	No. (%)		
	Riders (n = 228)^a^	Nonriders (n = 21)^a^	Total (N = 249)^a^
Triage acuity			
1: Most concerning	2 (0.9)	0	2 (0.8)
2	26 (11.4)	0	26 (10.4)
3	52 (22.8)	7 (33.3)	59 (23.7)
4	139 (61.0)	14 (66.7)	153 (61.4)
5: Least concerning	6 (2.6)	0	6 (2.4)
Missing^b^	3 (1.3)	0	3 (1.2)
Imaging			
Received any radiograph or CT	183 (80.3)	17 (81.0)	200 (80.3)
Received extremity radiograph or CT			
Upper extremity			
Distal	87 (38.2)	4 (19.0)	91 (36.5)
Proximal	39 (17.1)	3 (14.3)	42 (16.9)
Lower extremity			
Distal	47 (20.6)	3 (14.3)	50 (20.1)
Proximal	21 (9.2)	2 (9.5)	23 (9.2)
Received other radiography or CT^c^			
Chest radiograph	40 (17.5)	3 (14.3)	43 (17.3)
CT			
Head	66 (28.9)	8 (38.1)	74 (29.7)
Head and cervical spine	44 (19.3)	1 (4.8)	45 (18.1)
Head, cervical spine, chest, abdomen, and pelvis	21 (9.2)	0	21 (8.4)
Face	23 (10.1)	2 (9.5)	25 (10.0)
Cervical spine	45 (19.7)	1 (4.8)	46 (18.5)
Abdomen	22 (9.6)	0	22 (8.8)
Chest	21 (9.2)	0	21 (8.4)
ED length of stay for discharged patients^c^			
Patients discharged, No.	214	20	234
<4 h	156 (72.9)	19 (95.0)	175 (70.3)
>4 h	58 (27.1)	1 (5.0)	59 (23.7)
ED disposition			
Home	214 (93.9)	20 (95.2)	234 (94.0)
Admit to floor or observation	12 (5.3)	1 (4.8)	13 (5.2)
Intensive care unit	2 (0.9)	0	2 (0.8)
Injury characteristics^d^			
Any fracture	71 (31.1)	8 (38.1)	79 (31.7)
Upper extremity			
Distal	30 (13.2)	1 (4.8)	31 (12.5)
Proximal	15 (6.6)	2 (9.5)	17 (6.8)
Lower extremity			
Distal	9 (4.0)	2 (9.5)	11 (4.4)
Proximal	3 (1.3)	0	3 (1.2)
Facial	12 (5.3)	2 (9.5)	14 (5.6)
Vertebral column	2 (0.9)	0	2 (0.8)
Thoracic	3 (1.3)	1 (4.8)	4 (1.6)
Head injury	92 (40.4)	8 (38.0)	100 (40.2)
Minor head injury^e^	87 (38.2)	8 (38.0)	95 (38.2)
Intracranial hemorrhage	5 (2.2)	0	5 (2.0)
Contusions, sprains, and lacerations with no fracture or head injury	63 (27.5)	6 (28.6)	69 (27.7)
Dislocations			
Major^f^	9 (3.9)	0	9 (3.6)
Minor^g^	2 (0.9)	0	2 (0.8)
Procedural sedation for fracture reduction or joint dislocation	8 (3.5)	0	8 (3.2)
Lacerations	65 (28.5)	6 (28.6)	71 (28.1)
Major intra-abdominal or intrathoracic injuries^h^	3 (1.3)	0	3 (1.2)

Abbreviations: CT, computed tomography; ED, emergency department.

^a^ Unless otherwise noted.

^b^ 3 Cases were missing an acuity; on review, all 3 were trauma activations.

^c^ Proportions calculated based only on discharged patients.

^d^ Categories are not mutually exclusive.

^e^ Minor head injuries include all closed head injuries without skull fracture or intracranial hemorrhage.

^f^ Major dislocations include dislocations of the jaw, hips, shoulders, elbows, knees, and ankles.

^g^ Minor dislocations included dislocations of the fingers or foot.

^h^ Major intra-abdominal or intrathoracic injuries were defined as any internal injury of the thorax, abdomen, and pelvis represented by *International Classification of Diseases, Ninth Revision,* codes 860 to 869. The 3 cases included a splenic laceration and 2 lung contusions.

Among the 15 patients (6.0%) who were admitted or transferred, 13 patients were admitted to a floor or observation bed and 2 patients to the intensive care unit (one with traumatic subarachnoid hemorrhage, the other with a subdural hematoma). The reasons for hospitalization for the 15 patients admitted were orthopedic injuries (n = 5), intracranial hemorrhage (n = 5), major intra-abdominal or intrathoracic injuries (n = 3), cervical spine fracture (n = 1), and concussion (n = 1).

The most common injuries were fracture (79 patients [31.7%]), head injury (100 [40.2%]), and contusions, sprains, and lacerations without fracture or head injury (69 [27.7%]). Common fracture locations included the distal upper extremity (31 [12.5%]), proximal upper extremity (17 [6.8%]), distal lower extremity (11 [4.4%]), and face (14 [5.6%]). There was 1 open fracture. Eight patients (3.2%) received procedural sedation in the ED for reduction of a fracture or dislocation. Ninety-five patients (38.2%) sustained a minor head injury (head injury without intracranial hemorrhage or skull fracture), and 5 patients (2.0%) had an intracranial hemorrhage. Five of 95 patients (5.3%) with a minor head injury were documented as wearing a helmet during the incident, while none of the 5 patients with an intracranial hemorrhage had such documentation. Three patients had injuries to the intrathoracic or intra-abdominal organs, specifically pulmonary contusion, pneumothorax or hemothorax, and splenic injury.

A total of 193 scooter riders were observed during 3 public observation sessions, and the following unsafe riding practices were observed: no helmet use (182 riders [94.3%]), tandem riding (15 riders [7.8%]), and failure to comply with traffic laws (18 riders [9.3%]), as shown in eTable 2 in the Supplement. Additionally, many riders were observed to be riding on the sidewalk (51 riders [26.4%]), where scooter use is prohibited.

## Discussion

To our knowledge, this is the first study examining the injury patterns and clinical outcomes of patients presenting to the ED after incidents involving standing electric scooters. This rapidly expanding technology is a disruptive force in short-distance transportation, and policy makers seeking to understand associated risks and appropriate regulatory responses should seriously consider its effects on public health. Riders share roads with fast-moving vehicular traffic but appear to underestimate hazards; we found that 94.3% of observed riders in our community were not wearing a helmet. Unsurprisingly, injuries associated with standing electric scooter use are prevalent, with 249 patients presenting to the ED over the course of 1 year in our study of 2 EDs. Comparatively, in a post hoc analysis prompted by the review process, we identified 195 visits for bicyclist injuries (*ICD-10 *V10-V19) and 181 visits for pedestrian injuries (*ICD-10 *V00-V09) during the same time period at the 2 EDs. Scooter injuries documented in this study were mostly minor, but could also be severe and costly, with 6.0% of patients admitted to the hospital, and 0.8% admitted to the intensive care unit.

Like standing electric scooters, personal transporters launched by Segway offered a novel and convenient means of short-distance transportation, but came with a serious risk for orthopedic and neurologic trauma.^14,15,16^ Segway-related injuries commonly included upper and lower extremity fractures, but some were severe, including reported cases of intracranial hemorrhage requiring admission to the intensive care unit.^16^ We noted similar patterns of injury with standing electric scooters. However, unlike Segway transporters, standing electric scooters could have substantial impact on public health given their low cost, popularity, and accessibility.

While riders of electric scooters in California are required to be at least 16 years old by state law and 18 years old by company rental agreements,^17,18^ we found that 10.8% of electric scooter injuries were in patients younger than 18 years. This suggests that current self-enforced regulations imposed by private electric scooter companies may be inadequate. Although California law required helmet use while operating electric scooters during the entire study period, only 4.4% of injured scooter riders were documented to be wearing a helmet. A newly passed California law will make helmet use optional for electric scooter riders older than 18 years on January 1, 2019^11,12^; it is unclear how this change in policy will affect rider practices and injury patterns.

### Limitations

While this is the first study, to our knowledge, of trauma associated with electric scooter use to provide data on a full year of ED visits, our study is retrospective and therefore necessarily limited to available clinical variables. Future work would benefit from efforts to improve ED clinician documentation of relevant incident characteristics, such as helmet use. We likely underestimated the number of electric scooter–associated injuries for several reasons. We excluded 74 ED encounters where it was suspected, but not clear, that an electric scooter was involved, and we did not include outpatient visits to urgent care or primary care clinics for minor injuries. Additionally, scooter use and availability rapidly increased toward the end of our study period, evidenced by the fact that most associated injuries occurred during the later months of the study (eFigure in the Supplement). We were also unable to evaluate the geographic and urban planning factors influencing the incidence and severity of these injuries. Future work should include prospective data collection and examine the effects of bikeway availability and speed limits, which may modify the occurrence of injuries associated with electric scooter use. It would also be meaningful to characterize the costs incurred by patients and the health care system from trauma associated with electric scooter use. This descriptive study was unable to identify any risk factors for injury; future work could use data from private scooter companies to calculate the rates of injury based on number of trips, distance traveled, and demographic characteristics of scooter users.

## Conclusions

Standing electric scooters are a novel, innovative, and rapidly expanding form of transportation with the potential to alleviate traffic congestion, provide affordable transportation to residents of all incomes, and reshape how commuters travel the “last mile” to home or work. Our findings provide insight into the public health and safety risks associated with this rapidly growing form of transportation and provide a foundation for modernizing public policy to keep pace with this trend.

## Notes

Supplement. **eAppendix.** Supplemental Methods **eTable 1.** *ICD-9* Codes and Other Criteria Used to Generate Injury Categories **eTable 2.** Observation of Rider Behaviors and Pediatric Riders **eFigure.** Case Frequency by Date, Definite Cases (Included) vs Unclear Cases (Excluded)
