# Supplementary material for: Injuries Associated With Standing Electric Scooter Use
Source: JAMA Netw Open. 2019 Jan 25;2(1):e187381. doi: 10.1001/jamanetworkopen.2018.7381 (PMC6484536; doi:10.1001/jamanetworkopen.2018.7381)
Supplement: Supplement. — eAppendix. Supplemental Methods eTable 1. ICD-9 Codes and Other Criteria Used to Generate Injury Categories eTable 2. Observation of Rider Behaviors and Pediatric Riders eFigure. Case Frequency by Date, Definite Cases (Included) vs Unclear Cases (Excluded) [file jamanetwopen-2-e187381-s001.pdf]

Supplementary Online Content

Trivedi TK, Liu C, Antonio ALM, et al. Injuries associated with standing electric scooter use. *JAMA Netw Open*. 2019;2(1):e187381. doi:10.1001/jamanetworkopen.2018.7381

eAppendix. Supplemental Methods
eTable 1. ICD-9 Codes and Other Criteria Used to Generate Injury Categories
eTable 2. Observation of Rider Behaviors and Pediatric Riders
eFigure. Case Frequency by Date, Definite Cases (Included) vs Unclear Cases (Excluded)

This supplementary material has been provided by the authors to give readers additional information about their work.

© 2019 Trivedi TK et al. *JAMA Network Open.*

eAppendix: Supplemental Methods

Data Collection

The Office of Health Information Technology at UCLA provided 4 databases containing information for all ED encounters that met inclusion criteria; each contained a unique encounter number that could be used to link them to each other. All encounters occurred at Ronald Reagan UCLA Medical Center or UCLA Medical Center-Santa Monica between 9/1/2017-8/31/2018, and had to have at least one ED triage, nursing, or ED provider note that contained the terms “bird”, “lime”, or scooter”. We excluded encounters for *restricted patients*, defined as those who specifically asked that they never be included in research studies. Database 1 had one observation per encounter and contained the following variables: ED location, time of ED arrival, chief complaint, Emergency Severity Index (ESI) acuity level, mode of arrival, patient age, patient sex, ED disposition, ED length of stay, an indicator for trauma activation encounters, blood alcohol level if tested, hospital unit if admitted, and hospital length of stay. Database 2, 3, and 4 were in the “long” format; they contained multiple observations per encounter. Database 2 contained all triage, nursing, or emergency department provider notes associated with an encounter. Database 3 contained all orders for X-ray or CT imaging tests associated with each encounter. Database 4 contained all diagnoses assigned to each encounter.

Data Cleaning and Manipulation

Stata 15.0 was used for data cleaning and data summarization. Databases 2,3, and 4 were reshaped from a “long” format to a “wide” format, so that each individual observation represented a single encounter. For clarification, please refer to: https://stats.idre.ucla.edu/stata/modules/reshaping-data-long-to-wide/.

© 2019 Trivedi TK et al. *JAMA Network Open.*

First, all text fields in Database 2 (triage and provider notes) were replaced with lower case text. Next, each note variable was combined into one “combined note” variable, which then contained all text from all notes during that encounter. Each “combined note” was searched for the presence of the following terms “bird”, “lime”, “scooter”, “helmet”, “protective”, “electric”, “motorcycle”, “moped”, “vespa”, “non-motorized”, “wheelchair”, “segway”, and “razor”. Indicator variables were created for each term to facilitate manual review of the text entry data.

Each x-ray order in Database 3 was placed into one of the following imaging categories: proximal upper extremity (shoulder, clavicle, scapula), proximal lower extremity (elbow, forearm, wrist, hand, finger), distal lower extremity (knee, tibia/fibula, ankle, foot), proximal lower extremity (hip, femur, pelvis), chest X-ray, and other. CTs of extremities (e.g. elbow CT) were placed into the above categories given they represented a similar clinical concern for fracture or injury of that portion of the body.

Each diagnosis in Database 4 was placed into categories based on ICD9. All encounters had ICD9 and ICD10 diagnoses provided; we chose to analyze ICD9 given greater experience of using them in the trauma literature. There were a small number of patient encounters (N=7) in which ICD10 codes did not automatically map to a specific ICD9 code; for these encounters, we manually cross-referenced and assigned the matching ICD9 code. **eTable 1** provides the ICD9s used to generate the injury categories in Table 2.

Identification of cases

There were a total of 523 ED encounters with the terms “bird” “lime” or “scooter”. We used automatic free-text searching through Stata 15.0 to create indicator variables. Thus, manual chart review was assisted by indicator variables and by highlighting of the relevant terms listed above.

© 2019 Trivedi TK et al. *JAMA Network Open.*

An abstraction form was developed to capture determination of eligibility, reason for ineligibility if ineligible, described mechanism of traumatic event, documentation of helmet use, and documentation of intoxication. Two reviewers (TT and CL) split the cases in half and conducted manual chart review. Each reviewer piloted the initial abstraction form on 10 cases, and agreed on a final abstraction form. The two reviewers then independently reviewed the same 10 new cases, and agreed on eligibility and all other abstraction variables on all 10 of these cases.

For eligible cases, reviewers searched provider documentation for evidence of the use of a helmet (yes, no, unknown), documentation of evidence of intoxication in the history of present illness ("patient had been drinking alcohol", "patient admitted to being intoxicated", "patient smells of alcohol", etc.), and mechanism of accident (fall without additional details, scooter hit by moving vehicle, scooter hit a static object, pedestrian hit by scooter, pedestrian tripped over scooter, other). In many cases, the highlighted indicator variables made it immediately obvious that full chart review was not needed as the case could be excluded.

For a number of cases, it was clear that the encounter was for a standing electric scooter because the scooters were: 1) specifically referred to using an electric scooter company affiliation Bird or Lime (N=187) or 2) specifically referred to as "electric scooters" (N=69). All included cases met one of these two criteria.

In most excluded cases, it was obvious that the scooter was not the type of interest for this study and represented the sitting motorcycle scooters, electric mobility wheelchairs, or the non-motorized scooters popularized by the company Razor® that have been available for years.

© 2019 Trivedi TK et al. *JAMA Network Open.*

In 74 encounters, the term “scooter” was present, but it was unclear that the referenced scooters were the standing electric types. Because of the popularity of standing electric scooters and as many of these encounters were for patients aged 20-30, it is likely that at least some of these encounters were for standing electric scooter related injuries. However, these cases were not included in our results. eFigure 1 demonstrates the distribution of unclear cases and electric scooter cases by date over the one year study period.

Observational Study Methods

Four members of the research team observed the public common use patterns of standing electric scooter riders in two of the communities surrounding our hospitals (Santa Monica and Westwood, CA) for 7 hours between 9/8/2018 and 9/14/2018. The hours were chosen to include both weekday and weekend time periods and the intersections were chosen based on high use scooter areas previously observed in our communities. At a busy intersection in downtown Santa Monica, we stood at a corner and observed during a weekend afternoon between 2pm - 5pm (Location A: Promenade) and on a weekday morning between 10 am - 12pm (Location B: Promenade). At a similarly busy intersection on UCLA campus, we observed on a weekday morning between 11am - 1pm (Location C: UCLA campus). Observers only counted riders traveling in one direction and avoided counting the same rider twice.

Prior to initial rental using either Bird or Lime, riders agree to be over 18 years of age, to wear a helmet, to not “double ride” and to follow local laws. We recorded the following five characteristics of electric scooter use: helmet use, riding on sidewalks, riding two people to one scooter, rider with a likely visual age < 13 years and adherence to traffic laws. Though sidewalk riding on an electric scooter is variably legal depending on the community, and in fact many communities do not currently have laws directly related to electronic scooters on sidewalks as

© 2019 Trivedi TK et al. *JAMA Network Open.*

they are a new phenomenon, sidewalk riding likely poses a significant risk to pedestrians. We report summary statistics on the observational public use characteristics (eTable 2).

© 2019 Trivedi TK et al. *JAMA Network Open.*

eTable 1: ICD9 Codes and other criteria used to generate injury categories

Category	ICD9 Codes and Criteria
**Head Injury**	
Minor Head Injury	850, 854, 873, 959.01
Intracranial Hemorrhage*	851-853
**Any Fracture**	
Proximal Upper Extremity	810-812
Distal Upper Extremity	813-817
Proximal Lower Extremity	808, 820-821
Distal Lower Extremity	822-826
Facial	802
Vertebral Column	805, 806
Thoracic	807
**Contusions-Sprains-Wounds w/o Fracture or Head Injury**	If the encounter contained none of the above, and contained one of the following: 719-720, 840-848, 872, 874-879, 880-887, 890-897, 910-924, 959.1-959.9
**Major Dislocations**	830- 833, 835- 837
**Procedural Sedation for Fracture Reduction or Joint Dislocation**	Automatically identified using indicators for presence of the term "ketamine" "etomidate" "propofol" in occurrence with a fracture or dislocation. The notes of these 8 cases were manually checked to confirm procedural sedation was performed.
**Minor Dislocations (Fingers/Foot)**	834, 838
**Lacerations**	870 - 898
**Significant Intra-abdominal or Intrathoracic injury**	860-869

*One patient had code 432.1 (Nontraumatic subdural hemorrhage). However, in the clinical documentation, this was clearly documented as a traumatic subdural hemorrhage. We recorded this patient to 852.2. Seven patient encounters had ICD10 codes that did not automatically map to a specific ICD9 code. For these, we manually cross-referenced and assigned the matching ICD9 code.

© 2019 Trivedi TK et al. *JAMA Network Open.*

eTable 2: Observation of rider behaviors and pediatric riders.

	No Helmet Use	Sidewalk Riding	Breaking Traffic Laws	Double Riding	Pediatric Riders
Obs Period A (Weekend 2-5p, Location A: Promenade)	118 / 124 (95.2%)	22 / 124 (1.8%)	4 / 124 (3.2%)	10 / 124 (8.1%)	6 / 124 (4.8%)
Obs Period B (Weekday 10a-12p, Location B: Promenade)	24 / 28 (85.7%)	13 / 28 (46.4%)	7 / 28 (25%)	1/28 (3.57%)	0 / 28 (0%)
Obs Period C (Weekday 11a-1p, Location C: UCLA campus)	40 / 41 (97.6%)	16 / 41 (39.0%)	7 / 41 (17.1%)	4 / 41 (9.6%)	3 / 41 (7.3%)
Total	182 / 193 (94.3%)	51 / 193 (26.4%)	18 / 193 (9.3%)	15 / 193 (7.8%)	9 / 193 (4.7%)

© 2019 Trivedi TK et al. *JAMA Network Open.*

eFigure: Case frequency by date, Definite cases (Included) vs. Unclear Cases (Excluded)

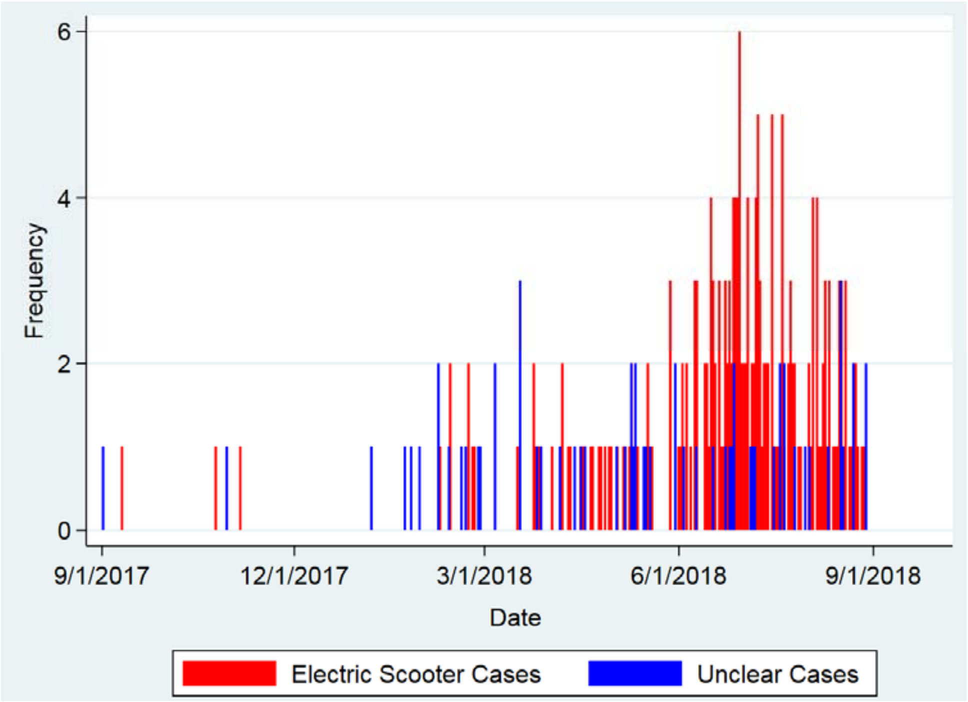

© 2019 Trivedi TK et al. *JAMA Network Open.*
